# A Case of Fournier's Gangrene in a Patient with Human Immunodeficiency Syndrome

**DOI:** 10.7759/cureus.76201

**Published:** 2024-12-22

**Authors:** Sahar S Abdelmoneim, Mariah K McIntyre, Milenis Lopez, Sohair Angly, Frontela Odalys

**Affiliations:** 1 General Internal Medicine, Larkin Community Hospital Palm Springs Campus, Hialeah, USA; 2 General Internal Medicine/Cardiovascular Medicine, Assiut University Hospitals, Asyut, EGY; 3 General Internal Medicine, Larkin Community Hospital South Miami Campus, South Miami, USA

**Keywords:** adult hiv, fournier's gangrene, fournier's gangrene prognosis and treatment, hiv-associated infection, human immunedeficiecy virus (hiv) infection, obesity and necrotizing fasciitis, pocus (point-of-care ultrasound), sub-saharan africa hiv

## Abstract

Fournier’s gangrene (FG) is a type of necrotizing fasciitis affecting the abdomen or perineum. It is a polymicrobial infection that progresses to an obliterating endarteritis, causing thrombosis and subsequent tissue necrosis, allowing pathogenic invasion of interfacial planes.Patients with Fournier’s gangrene typically have underlying systemic conditions that cause vascular insufficiencies or immunosuppression. This case describes a 55-year-old male patient with a medical history significant for HIV, obesity (BMI of 37), and smoking. The patient presented with generalized symptoms along with localized edema and erythema of the scrotum. Point-of-care ultrasound (POCUS) aided in evaluating the deeper structures for accurate diagnosis of Fournier’s gangrene. Treatment followed current standards, including empiric antibiotics, analgesia, and surgical incision and drainage. The patient experienced an uncomplicated postoperative recovery despite his comorbidities due to prompt diagnosis and treatment.

## Introduction

The development of necrotizing fasciitis in the abdomen or perineum is known as Fournier's gangrene (FG). It is a polymicrobial infection, typically involving an average of four organisms, that progresses to an obliterating endarteritis. This endarteritis leads to vascular thrombosis and subsequent tissue necrosis, allowing pathogens to migrate into traditionally sterile interfacial planes [1,2]. Thrombosis commonly occurs in the perineal branches of the internal pudendal artery for infections of perineal origin. When the infection originates in or extends to the abdomen, thrombi are more frequently found in the deep circumflex iliac artery and superficial inferior epigastric artery. While the scrotum is most often affected, the testicles are typically spared as they are supplied more directly by the aorta [3,4]. The resulting thrombosis causes ischemia in the invaded tissues, which leads to necrosis and creates an environment conducive to the proliferation of anaerobic bacteria. The most commonly identified pathogen is *Escherichia coli*, followed by *Streptococcus*. Other contributing pathogens include *Bacteroides*, *Enterobacter*, *Staphylococcus*, *Enterococcus*, *Pseudomonas*, *Corynebacterium*, and *Klebsiella pneumoniae *[5].

There is a male preponderance in the incidence of FG, though 10% of cases occur in women [6]. FG typically presents in men between the ages of 50 and 79 years, with an overall incidence of 1.6 per 100,000, accounting for 0.02% of annual hospital admissions in the United States [7]. FG is associated with a high mortality rate, ranging from 20% to 30%. The most common sign of FG, which progresses slowly, is scrotal swelling. Other related symptoms include fever, tachycardia, subcutaneous emphysema, and pus-like exudation leaking from the perineum [8]. Given its often insidious course and significant mortality, FG must remain a differential diagnosis for early identification and prompt treatment. 

Patients with FG typically have systemic conditions that cause vascular insufficiencies or immunosuppression, which increases their susceptibility to infection [3]. Contributing risk factors include various chronic disease comorbidities, with human immunodeficiency virus (HIV) infection being particularly significant, as it adversely affects the prognosis and likelihood of developing FG [8]. The occurrence and severity of FG seem to be rising in parallel with the increasing prevalence of HIV [9].

The purpose of presenting this case is to highlight the incidence of FG in HIV patients and to emphasize the importance of considering FG in the differential diagnosis for acute scrotum, as early intervention is critical to improving survival and quality of life after surgical debridement.

## Case presentation

**Figure 1:**
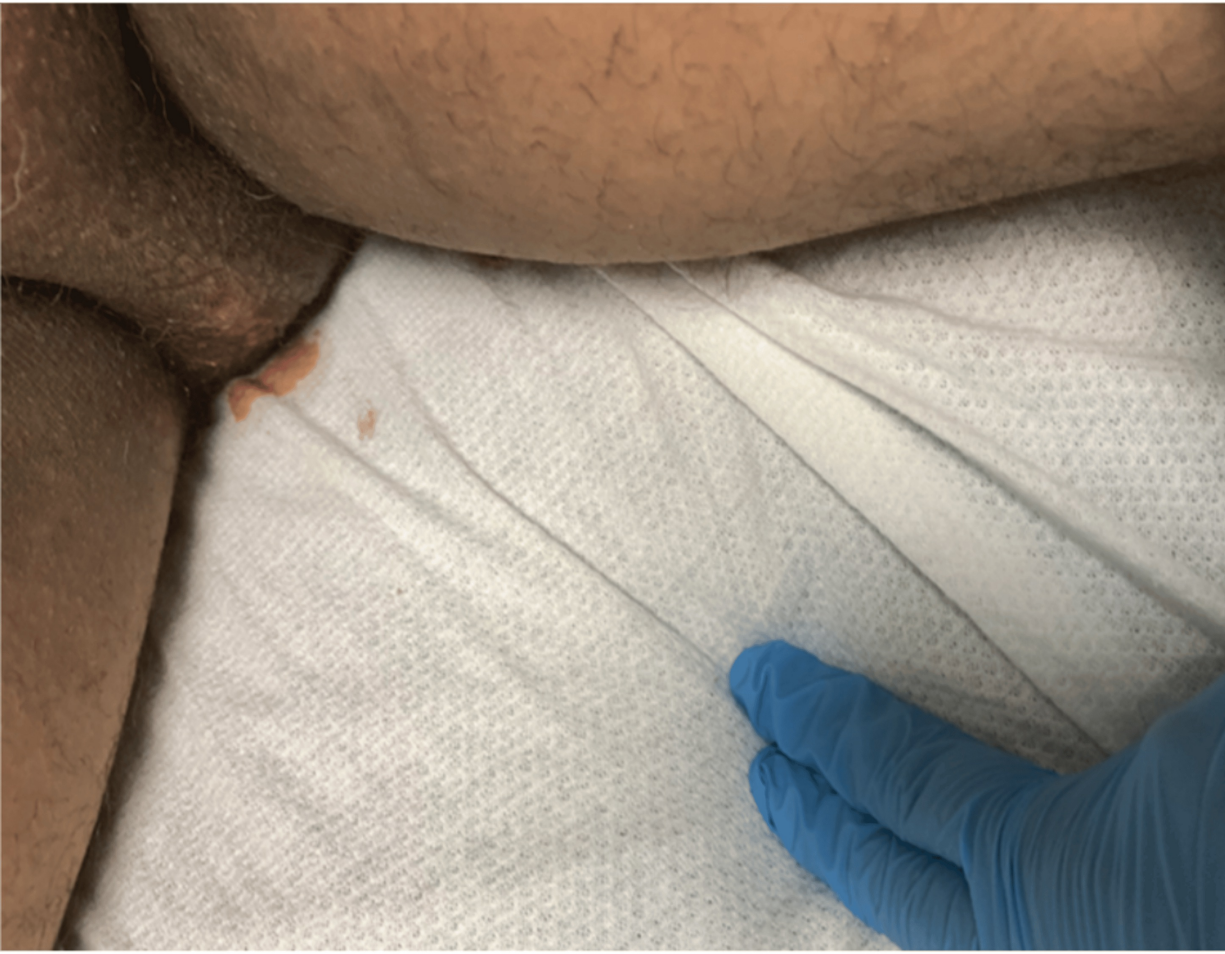
Male genital examination An abnormal erythematous scrotal skin color, edema, and tender indurated swelling with oozing fluid were evident.

**Figure 2:**
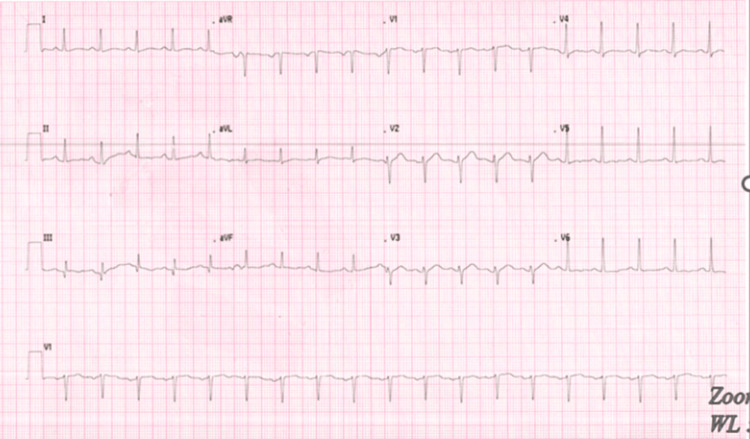
Electrocardiogram on admission showing sinus tachycardia rate (114 beats per minute)

**Figure 3:**
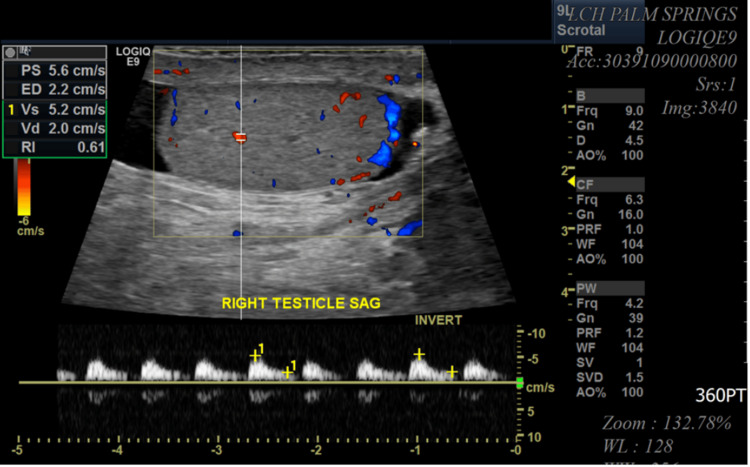
US exam of the testes and perineum showing normal-sized right testicle

**Figure 4:**
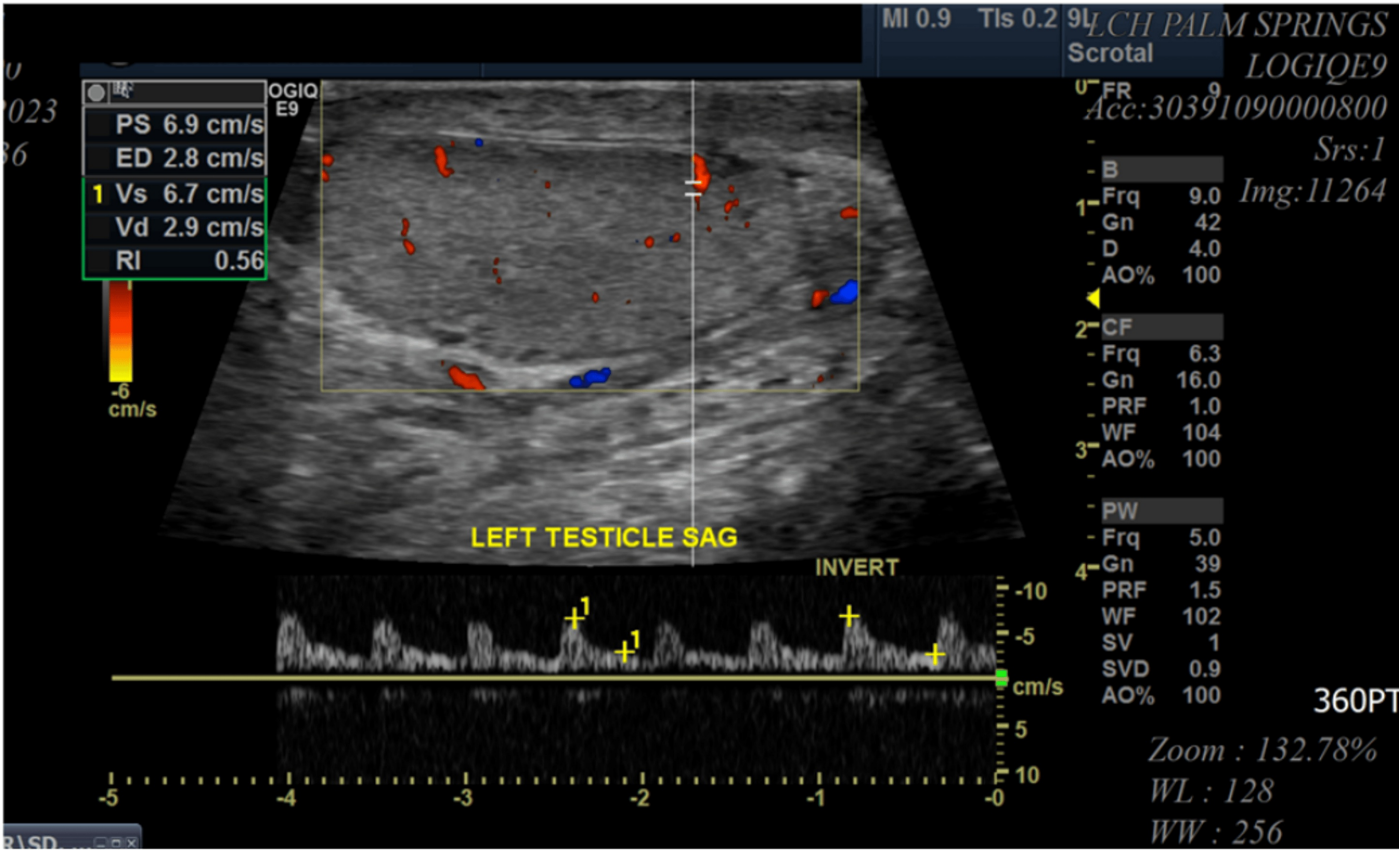
US exam of the testes and perineum showing normal-sized left testicle

**Figure 5:**
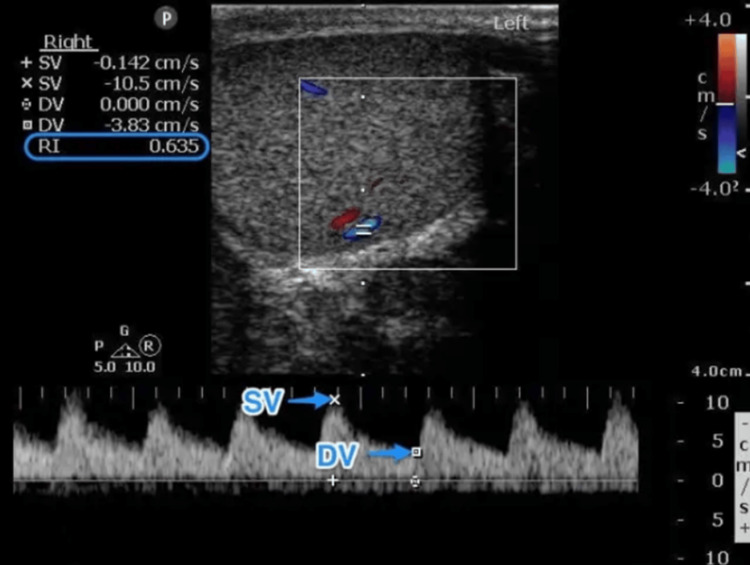
US exam of the testes and perineum showing edema in the perineum Normal resistance index (right, 0.56; left, 0.61).

**Figure 6:**
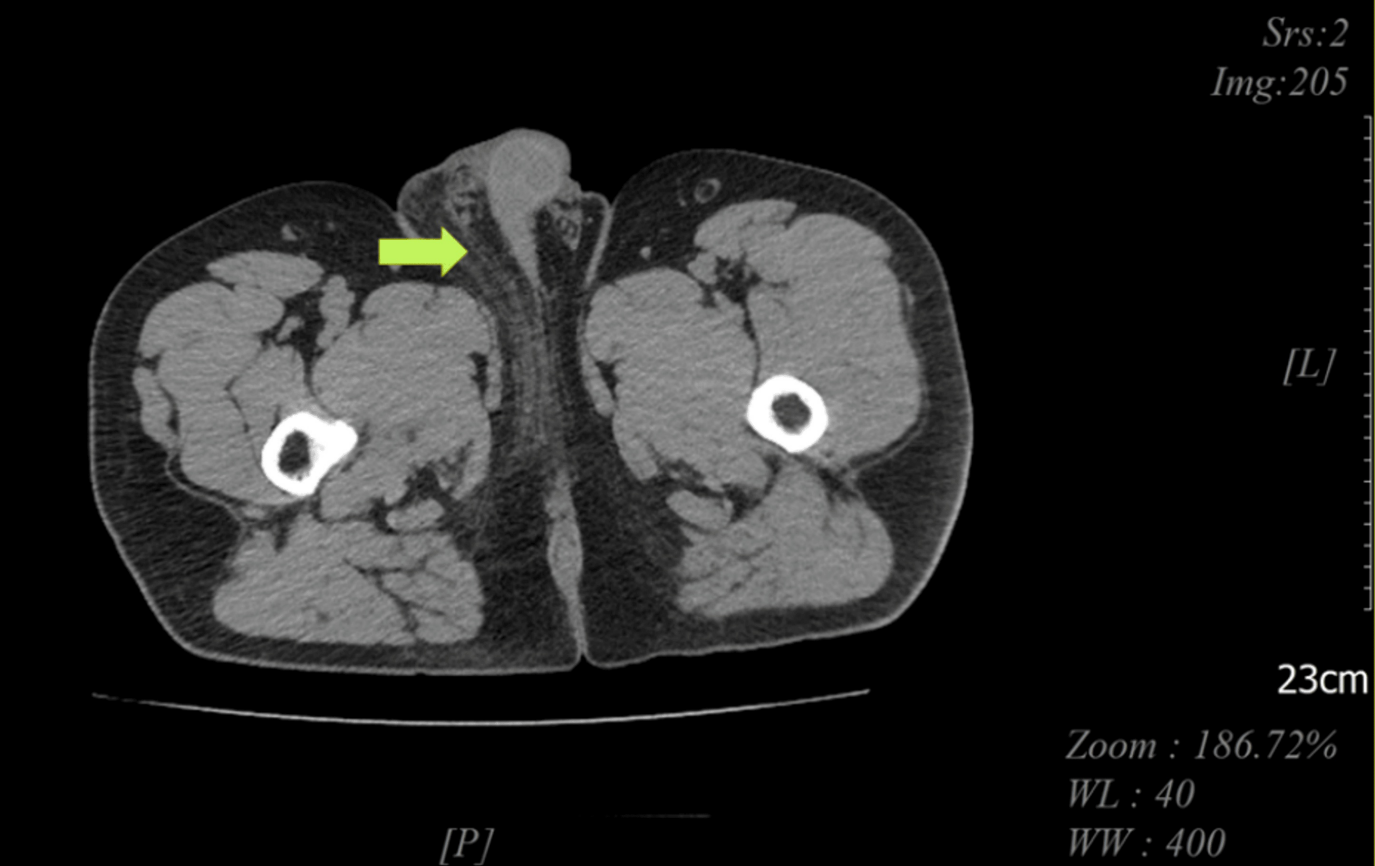
Abdominal/pelvic CT showing fat stranding in the perineum more evident on the right side than on the left (green arrow).

A 55-year-old male patient with a significant medical history of HIV, obesity (BMI of 37), and heavy smoking presented to the emergency department with pain in the perianal and groin regions. The onset of his symptoms occurred two days before his presentation but worsened significantly over the past 24 hours. The pain was described as dull, widespread, and consistent, with an intensity rating of 10/10. The patient also reported chills, fever, and scrotal swelling. There was minimal relief of pain with scrotal elevation. In the emergency room, the patient was afebrile (99 °F) and tachycardic (heart rate of 114 beats per minute), with blood pressure of 138/67 mmHg, breathing rate of 18 breaths per minute, and 100% pulse oximetry on room air. His genitourinary examination revealed erythematous, edematous scrotal skin with tender indurated swelling but no penile or urethral discharge or anal discharge (Figure 1). No rashes, itching marks, sharply demarcated borders, or fluctuation were noted. The rectal exam was not remarkable. The cremasteric reflex was negative. The physical exam was otherwise unremarkable. A sinus tachycardia was noted on the admission electrocardiogram, as shown in Figure 2. Laboratory analysis was significant for blood glucose of 131 mg/dL (74-106 mg/dL), hemoglobin A1C of 6.2%, lactic acid of 1.5 mmol/L (0.7-2.1 mmol/L), and white blood cell count of 18 K/µL (3.8-9.8 K/µL). Urinary analysis (UA) was unremarkable, apart from a few bacteria. CD4 count was 750 cpm (49%), and viral load was undetectable. The patient underwent a point-of-care ultrasound (POCUS) that demonstrated interruption of the muscle fascial planes and the presence of subcutaneous gas. On ultrasound, bilateral testicles showed normal-sized right and left testicles with normal resistive index (RI of 0.61 and 0.56, respectively), as illustrated in Figures 3-5. An abdominal/pelvic CT showed evidence of fat stranding of the perineum (right > left) and hepatic steatosis, as shown in Figure 6. Given the patient's clinical presentation, leukocytosis, skin examination, and imaging findings, the diagnosis of necrotizing fasciitis (FG) was made. The patient was immediately started on normal saline 0.9% 1000 mL bolus twice, empiric antibiotics (intravenous (IV) vancomycin 1000 mg every 24 hours, and piperacillin-tazobactam 3.375 gm IV every 12 hours), and pain control (acetaminophen 650 mg PO every six hours). A surgical consult was placed for possible intervention. Urine and blood cultures showed no growth after 48 hours and up to five days, respectively. As the patient's condition evolved, skin changes were noted, including pus discharge from a small opening on the top of the swelling. This led to the patient undergoing incision and drainage. The patient's hospital course was uneventful. 

## Discussion

FG is a rare form of necrotizing fasciitis of the genitalia or perineum, likely polymicrobial in etiology [2]. FG usually occurs as a consequence of surgical site infections, pressure sores, or vaginal or anorectal abscesses. Increased susceptibility for severe FG was shown to be more frequent in obese patients with HIV or diabetes mellitus [8,9]. Patients with FG may present with nonspecific symptoms, while others are asymptomatic [6]. 

This case highlights the critical need for prompt diagnosis of FG to prevent potentially life-threatening complications. The foundational therapeutic strategies for FG include sufficient hemodynamic resuscitation, urgent surgical debridement, and the prompt initiation of broad-spectrum parenteral antibiotics. The majority of FG-related mortality is attributed to sepsis, which further substantiates the necessity for early recognition and intervention. Studies have shown that patients with HIV experience similar mortality rates when treatment is expedited [10]. In our case, POCUS played a key role in rapidly establishing a working diagnosis. The identification of gas within the fascial and muscular planes via POCUS was crucial for diagnosing FG. POCUS has been recognized as a quicker, more accessible alternative to gold-standard CT imaging, especially in busy hospital settings or for unstable and critically ill patients [11]. FG often presents deceptively with minimal outward signs, as most of the damage occurs between the fascial and muscular layers. This can lead to delayed diagnosis and treatment. Given the rising prevalence of diabetes, obesity, and HIV in the United States, it is increasingly important to consider FG early in the differential diagnosis for patients presenting to the emergency room, as early intervention is paramount to preventing complications, morbidity, and mortality associated with incision, drainage, and debridement [12].

Table 1 presents a selected review of the literature on cases presenting with FG and their clinical outcomes. 

**Table 1:** Selected review of literature of cases presenting with Fournier's gangrene and their clinical outcomes HIV: human immunodeficiency virus, DM: diabetes mellitus, FG: Fournier's gangrene.

Authors	Country	Study population	Treatment	Results and conclusion
Merino et al. [2]		Medline database search for all English literature with cases of FG in HIV patients from 1980 to 2000. A total of 12 cases were included.	Urgent surgical debridement and intravenous broad-spectrum antimicrobials.	Nine of 12 (75%) with CD4+ cell count < 200 μL/cells had AIDS prior to the development of FG. The authors concluded that severe immunosuppression increases the risk of FG, with a minor role in CD4+ cell depletion.
Chalya et al. [6]	Tanzania	A total of 84 patients with FG.	Broad-spectrum antibiotics (meropenem and imipenem) and wide surgical excision.	Main prognostic factors of mortality included elderly, diabetes mellitus, HIV with low CD4 (<200 μL/cells), late symptomatology, infection involving the abdominal wall, and high Fournier’s gangrene severity score (>9).
Lewis et al. [8]	United States, Grenada	N/A. Meta-analysis.	Emergent surgical intervention with debridement, broad-spectrum antibiotics, and resuscitation with intravenous (IV) fluids.	Perineal pain, cellulitis, fever, abscesses, and crepitus were the main clinical presentations.
Ngugi et al. [9]	Kenya	A total of 146 male patients treated for FG.	Empirical broad-spectrum antibiotics are based on culture and sensitivity.	Twenty-one percent of patients died, with sepsis being the most common cause of death in 90% of cases. Comorbidities included HIV, diabetes, and alcoholism.
Elem and Ranjan [10]	Zambia	A total of 10 patients with an average age of 32 years were followed during a 14-month period. Eight of 10 patients had HIV.	Surgical debridement and triple antibiotic regimen (gentamicin, metronidazole, and penicillin).	Despite all patients having HIV, it did not adversely affect the prognosis of Fournier's gangrene.
Elsaket et al. [12]	South Africa	A total of 44 patients during a five-year study period.	Forty-three of 44 patients (98%) underwent surgical debridement. Broad-spectrum antibiotics included amoxiclav, metronidazole, and gentamicin.	HIV patients presented with FG at a younger age compared to non-HIV patients. However, neither HIV nor DM was significantly associated with mortality.

## Conclusions

This case highlights the critical need for prompt diagnosis of FG to prevent potentially life-threatening complications. The primary therapeutic strategies for FG include effective hemodynamic resuscitation, urgent surgical intervention, and the immediate initiation of broad-spectrum parenteral antibiotics. The majority of FG-related mortality is attributed to sepsis, which further substantiates the necessity for early recognition and intervention. In our case, POCUS played a key role in rapidly establishing a working diagnosis. The identification of gas within the fascial and muscular planes via POCUS was crucial for diagnosing FG. FG often presents deceptively with minimal outward signs, as most of the damage occurs between the fascial and muscular layers. This can lead to delayed diagnosis and treatment. Given the rising prevalence of diabetes, obesity, and HIV in the United States, it is increasingly important to consider FG early in the differential diagnosis for patients presenting to the emergency room, as early intervention is paramount to preventing complications, morbidity, and mortality associated with incision, drainage, and debridement.
